# E-learning pills on immunotherapy in urothelial carcinoma: The E-PIMUC program for continuing medical education

**DOI:** 10.3389/fphar.2024.1380954

**Published:** 2024-08-22

**Authors:** Olga Romero-Clarà, Clara Madrid, Juan Carlos Pardo, Vicenç Ruiz de Porras, Olatz Etxaniz, Deborah Moreno-Alonso, Albert Font

**Affiliations:** ^1^ e-oncología Online Training Unit, Catalan Institute of Oncology, L’Hospitalet de Llobregat, Barcelona, Spain; ^2^ Medical Oncology Department, Catalan Institute of Oncology, Barcelona, Spain; ^3^ Badalona Applied Research Group in Oncology (B·ARGO), Catalan Institute of Oncology, Barcelona, Spain; ^4^ CARE program, Germans Trias i Pujol Research Institute (IGTP), Barcelona, Spain; ^5^ GRET and Toxicology Unit, Department of Pharmacology, Toxicology and Therapeutic Chemistry, Faculty of Pharmacy and Food Sciences, University of Barcelona, Barcelona, Spain

**Keywords:** urothelial carcinoma, bladder cancer, immunotherapy, healthcare professionals, continuing medical education, microlearning, e-learning pills

## Abstract

**Background:**

The high incidence and mortality rates of urothelial carcinoma mean it remains a significant global health concern. Its prevalence is notably pronounced in industrialized countries, with Spain registering one of the highest incidences in Europe. Treatment options are available for various stages of bladder cancer. Moreover, the management landscape for this disease has been significantly transformed by the rapid advances in immunotherapy. Healthcare professionals who diagnose, treat, and follow up with bladder cancer patients need comprehensive training to incorporate these advances into their clinical practice. To bridge these knowledge gaps, we set up the E-PIMUC program to educate healthcare professionals on bladder cancer management and specifically immunotherapy.

**Methods:**

E-PIMUC used an innovative microlearning methodology comprising bitesize learning pills that support efficient acquisition of specialized expertise. We used a mixed methods, quantitative and qualitative approach to assess the success of the E-PIMUC program. Data collection encompassed pre-post testing, participation metrics, satisfaction surveys, and self-perceived performance assessments.

**Results:**

A total of 751 healthcare professionals enrolled in the program. Of these, 81.0% actively engaged with the content and 33.2% passed all tests and were awarded the course certificate and professional credits. The course received satisfaction ratings of 94.3% to 95.1% and significantly improved the declarative knowledge of participants who had a range of professional profiles (*p* < 0.001). Participants reported increased confidence in applying immunotherapy principles in their practice (average improvement of 1.4 points). Open-ended responses also underscored participants’ perceived benefits, including expanded knowledge and enhanced patient interaction skills.

**Conclusion:**

The E-PIMUC program provided effective, comprehensive, cutting-edge training on bladder cancer management, particularly on the use of immunotherapy in this area of oncology. The high participation rates, positive satisfaction scores, substantial knowledge enhancement, and improved self-perceived performance, are all testament to the program’s success. E-PIMUC was endorsed by regulatory bodies as a trusted educational resource in urothelial carcinoma management. What is more, complementary initiatives brought together patients and medical experts to foster a holistic, patient-centered approach to the complexities of bladder cancer care.

## 1 Introduction

### 1.1 Immunotherapy and urothelial carcinoma

Bladder cancer remains a significant global health concern due to its high incidence and mortality rates. It is the ninth most prevalent cancer worldwide, with an annual incidence of 430,000 cases. Among men, bladder cancer is the fourth most common cancer after lung, prostate, and colon cancer. Bladder cancer is the tenth most frequent cause of cancer-related death globally, ranking eighth among men and fourteenth among women (Jubber et al., 2023; Siegel et al., 2021). Incidence is especially high in industrialized countries, including Spain, which has the fourth highest incidence of bladder cancer in Europe – 27 cases per 100,000 inhabitants – and the second highest estimated mortality (Sung et al., 2021).

The 70% of patients who present at initial diagnosis with non-muscle-invasive bladder cancer can usually be managed with local treatment strategies and subsequent medical follow-up. By contrast, tumors invading the detrusor muscle are known as muscle-invasive bladder cancer. These account for 20% of bladder cancer cases, and 15%–20% of superficial bladder cancer progression to muscle-invasive bladder cancer. The standard treatment for muscle-invasive bladder cancer is perioperative cisplatin chemotherapy followed by cystectomy (Ruiz de Porras et al., 2022). For the approximately 5% of patients who present with metastatic bladder cancer at diagnosis, platinum-based chemotherapy is also currently considered the gold standard of treatment (Bellmunt et al., 2022).

The advent of immunotherapy has caused a transformative shift in how cancer and particularly blader cancer is treated. The use of immune checkpoint inhibitors to treat advanced bladder cancer has substantially improved overall survival and quality of life (Bellmunt et al., 2022; Galsky et al., 2021). Immune checkpoint inhibitors also provide sustained benefits for patients treated previously with platinum-based first-line chemotherapy (Powles et al., 2020). This success has prompted the exploration of immunotherapy interventions at earlier stages of bladder cancer, including patients with muscle-invasive bladder cancer (Ruiz de Porras et al., 2022). For the latter group, the anti-PD-1 agent nivolumab was approved as adjuvant treatment after cystectomy (Bajorin et al., 2021). Extensive research has also been undertaken to identify molecular and histological biomarkers in tumor tissue and liquid biopsies to enable targeted patient selection for immunotherapy-based treatments (Luceno et al., 2023; Meeks et al., 2023; Crupi et al., 2023).

### 1.2 E-learning pills: an innovative solution to bridge a critical knowledge gap

Healthcare professionals who diagnose, treat, and follow up with cancer patients need comprehensive training to keep pace with advancements in immunotherapy. In 2018, our team conducted the VIKHI project^1^ (Virtual International Knowledge Hub in Immuno-therapy) to evaluate immunotherapy knowledge among 150 clinicians and nurses across Spain, the United Kingdom, and Italy, and to identify their specific educational needs. The analysis revealed significant gaps: 46% of respondents did not know the mechanism of action of immune checkpoint inhibitors, over 40% were unaware of why certain solid tumors respond to these inhibitors or how to evaluate tumor response to immunotherapy, and between 15% and 53% lacked confidence in managing immunotherapy toxicities. Moreover, a substantial number of respondents were unfamiliar with clinical guidelines on immune-related adverse events, and over 90% expressed a desire for specific training on immunotherapy. We uncovered significant knowledge gaps concerning immunotherapy mechanisms, how to assess tumor responses, and how to effectively manage adverse events. These deficiencies suggested an urgent need for targeted educational interventions.

The “E-learning Pills on Immunotherapy in Urothelial Cancer” (E-PIMUC) project^2^ was our response to this unmet training need. The program was accredited by the Catalan Council of Continuing Education for Healthcare Professionals (CCFCPS)^3^ and is worth 2.4 professional credits. Using an innovative microlearning methodology, E-PIMUC offered a series of e-learning pills on immunotherapy in urothelial cancer catering to specific educational needs. The learning pills respond to the needs of a busy modern society by imparting knowledge in small, targeted doses to support rapid acquisition of expertise. They contain comprehensive content on immunotherapy mechanisms, adverse event recognition, and treatment response assessment, and directly target the knowledge gaps we identified in the VIKHI project. E-PIMUC accommodates diverse learning styles and includes multimedia material, interactive elements, and gamification, all of which ensures user engagement and facilitates better understanding and retention of complex medical concepts among healthcare professionals working in bladder cancer care.

There is evidence in the literature supporting microlearning and tailored online training in diverse educational contexts. A recent comparison of microlearning vs. traditional e-learning found that evaluation completion rates were slightly higher with the microlearning approach (Bannister et al., 2020). A separate analysis of the effectiveness of microlearning continuing medical education (focusing in this case on recent developments in early and metastatic breast cancer) revealed a statistically significant enhancement in knowledge, proficiency, and confidence levels among participants who completed the assessments (Dewji et al., 2023). Digital microlearning may also enhance feedback competencies among healthcare providers (Safavi et al., 2022).

Specific training programs for urologists and oncologists on clinical management of immune checkpoint inhibitors in urothelial cancer have yielded positive outcomes, including increased confidence to implement immunotherapy and to manage adverse events (Gitzinger et al., 2021; Gitzinger et al., 2022). However, to the best of our knowledge none of these programs used a microlearning-based methodology.

In this paper, we comprehensively analyze the E-PIMUC program and evaluate its impact on participants’ knowledge and satisfaction. Our methodology is based on an adapted evaluation framework inspired by Moore, Green, and Gallis, encompassing four key dimensions: participation, satisfaction, learning, and performance (Moore et al., 2009). Our findings offer valuable insights that have wider implications for continuing medical education.

## 2 Materials and methods

### 2.1 Study design

Our mixed methods approach comprised a single group, pre-post study with a combination of quantitative and qualitative evaluation instruments and methods to collect data for all levels of our adapted version of the evaluation framework created by Moore, Green, Gallis (Moore et al., 2009).

### 2.2 Participants

751 professionals enrolled in E-PIMUC. Of these, 608 (81.0%) accessed the program content and 249 (33.2%) passed all tests, received the course certificate, and were awarded professional credits (Table 1).

**TABLE 1:** Enrollment, participation, and accreditation rates for the E-PIMUC program.

	Registered participants		Accessed the content		Completed the full program	
	N	%	n	%	n	%
Total	751	100	608	81.0	249	33.2

### 2.3 E-PIMUC content

E-PIMUC was a comprehensive educational initiative with the primary objective of enriching the knowledge and skills of healthcare professionals who manage urothelial cancer patients. The flexible and accessible learning format is built around microlearning pills designed specifically for the busy healthcare professional, providing a small dose of new or updated knowledge to refresh and boost participants’ skills in an efficient manner. Microlearning, a key feature of our approach, delivers content in brief, targeted modules typically lasting 10–15 min. This format contrasts with traditional e-learning, which tends to cover broader topics over longer periods, often spanning hours or days.

The instructional design of the E-PIMUC program is grounded in cognitive learning theories such as Piaget’s constructivism, Ausubel’s meaningful learning, Bruner’s scaffolding, and Vygotsky’s social constructivism. This theoretical foundation supports our innovative approach of delivering content through “learning pills”, which are concise, engaging modules designed to optimize knowledge retention and application among healthcare professionals. The incorporation of gamification further enhances learning by fostering active engagement and motivation.

The program comprised three modules:

• Module 1: Treatment of Urothelial Carcinoma: Current Status and Future Perspectives (4 h)
• Module 2: Molecular Biology and Predictive Biomarkers (3 h)
• Module 3: Clinical Management of Urothelial Carcinoma (4 h)

**FIGURE 1:**
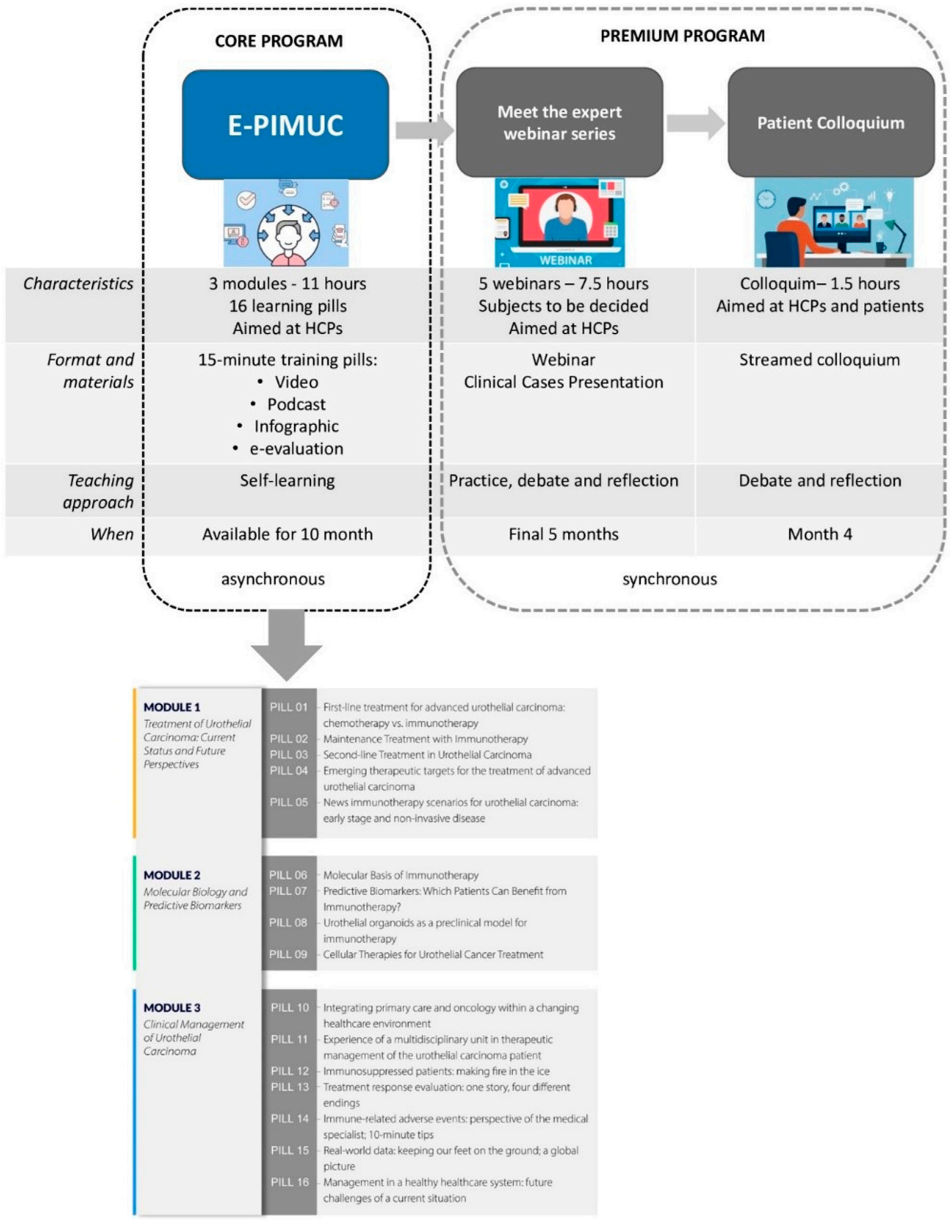
E-PIMUC program contents.

Each module is divided into sections that are further broken down into digestible units called learning pills (Figure 1). The average learning pill duration is 15 min and the longest is 45 min. Since each learning pill can be studied independently at any time, users can tailor the course to suit their own needs.

The development process included:

a) Detection phase: An analysis of training needs relative to bladder cancer treatment to determine which microcontent to develop through a survey of 50 professionals from the target audience.
b) Construction phase: We worked with 22 highly qualified experts across the different E-PIMUC topics to create the learning pills. Their experience and expertise in each area of the program ensured that participants obtained accurate, comprehensive, cutting-edge training. This phase also included: refining the content to address identified needs and meet set objectives, and producing the training pills in video, podcast, and infographic formats.

Each of the 16 e-learning pills addresses a specific topic within the corresponding module. A total of 57 video lessons together with other resources and interactive activities comprise over 10 h of training content.

To further enhance the learning experience, E-PIMUC users who completed the program were offered the opportunity to join a series of three complementary webinars featuring a panel of clinical experts. The webinars delved deeper into specific topics covered in the microlearning pills, fostering in-depth discussion and addressing participants’ questions. In addition, a virtual colloquium titled *The urothelial carcinoma patient: Experience, needs, concerns, and expectations* promoted inclusive dialogue between patients and healthcare professionals.

E-PIMUC sets itself apart from NEJM (New England Journal of Medicine) and ASCO (American Society of Clinical Oncology)’s microlearning platforms by offering customized educational materials designed for Spanish-speaking professionals in Spain and Ibero-American nations. In contrast to NEJM and ASCO, E-PIMUC incorporates interactive features such as the “Meet the Expert Webinar Series” and “Virtual Patient Colloquium,” enabling meaningful interactions with specialists and patients to explore urothelial cancer treatment. While NEJM and ASCO serve as valuable reference points, E-PIMUC stands out for its tailored needs assessment, regional and language-specific focus, and supplementary elements that underscore the efficacy of microlearning in bridging knowledge gaps and enhancing education for professionals and patients alike.

### 2.4 Data collection

Our adapted version of the Moore, Green, and Gallis evaluation framework included four of the seven original assessment levels. We also added a level 0 for identifying participants’ learning needs prior to the course:

*Training Needs Assessment (Level 0)*: we disseminated a learning needs survey among healthcare professionals, including members of scientific societies (e.g., the Spanish Oncology Genito Urinary Group^4^, and the Spanish Medical Oncology Society^5^) and ICO staff. The survey probed the motivations for participating in the program and identified respondents’ priority training needs around immunotherapy in bladder cancer.

*Participation (Level 1)*: participation metrics were collected from learning management system registration data, including the number of registered participants and demographics such as profession, specialty, organization, and location. We also tracked engagement metrics: content access, activity completion, and course completion times.

*Satisfaction (Level 2):* participants received post-course satisfaction surveys that delved into their expectations and their satisfaction with respect to how the course was organized, the methodology, and the content. We also assessed participants’ perceived applicability of the training program.

*Learning and Performance (Levels 3 and 5)*: we conducted pre-post testing to assess participants’ declarative knowledge (Level 3) regarding the program content and their self-perceived performance (Level 5) in terms of knowledge transfer and usefulness.

Due to constraints in resources—financial, human, and time—we did not explore Levels 4 (behavior change), 6 (organizational impact), and 7 (community impact). These levels require extensive longitudinal studies that were beyond the scope of our 18-month project.

### 2.5 Data Analysis

Registration data and engagement metrics underwent statistical analysis. We used Microsoft Excel 2021 to calculate descriptive statistics such as means, standard deviations, and percentages.

Data analysis from Level 3 and 5 questionnaires was performed using SPSS (version 23). We used the Shapiro-Wilk test to determine if the data followed a normal distribution pattern, since this test is suitable for smaller sample sizes. The results indicated a deviation from normality in the self-assessment scores for knowledge (Level 3) and performance (Level 5) both before and after the training sessions, so we used nonparametric statistical methods for subsequent analyses to ensure robustness in the assessment. We applied the Wilcoxon signed-rank test to compare self-assessments of knowledge and performance before and after the course. A *p*-value less than 0.05 was considered statistically significant. Missing data (incomplete questionnaires) were excluded from the analysis.

Answers to open-ended questions underwent thematic analysis to identify and group recurring themes, patterns, and participants’ qualitative insights around the perceived benefits of the course.

## 3 Results

### 3.1 Level 1 – Participation

The 608 professionals who actively engaged in the program – including accessing a specific learning pill that matched their interests or needs – had diverse professional profiles, specialties, organizational affiliations, and prior training, with the vast majority of participants (76.8%) being nurses. Their specialties included oncology nursing (43.8%), medical oncology (22.4%), and to a lesser degree primary care (10.7%), with smaller groups coming from other areas. Around two thirds of participants were affiliated with hospitals. Remarkably, over three quarters of healthcare professionals who actively engaged with the content lacked any prior training, and almost all lacked any specific training in managing urothelial carcinoma (Tables 2–5).

**TABLE 2:** Participation by professional profile (Level 1. Participation).

Profile	Registered participants	Accessed the content		Completed the full program	
		n	%	n	%
Nurse	557	467	83.8%	187	33.6%
Physician	123	85	69.1%	35	28.5%
Researcher	26	19	73.1%	8	30.8%
Pharmacist	45	37	82.2%	19	42.2%
All	751	608	81.0%	249	33.2%

**TABLE 3:** Professionals who actively engaged in the program: specialty (Level 1. Participation).

*Speciality*	Participants who accessed the content	
	n	
Medical Oncology	136	22.4%
Radiation Oncology	16	2.6%
Primary Care	65	10.7%
Palliative care	15	2.5%
Internal Medicine	37	6.1%
Pharmacy	37	6.1%
Hematology	17	2.8%
Research	19	3.1%
Oncology Nursing	266	43.8%

**TABLE 4:** Professionals who actively engaged in the program: organizational affiliation (Level 1. Participation).

*Organisational affiliation*	Participants who accessed the content	
	n	%
Hospital	408	67.1%
Cancer monographic center	38	6.3%
Private center	41	6.7%
Primary Care Center	51	8.4%
Research Institute	19	3.1%
Other	51	8.4%

**TABLE 5:** Professionals who actively engaged in the program: prior training (Level 1. Participation).

*Prior training*	*In inmunotherapy*		*In management of urohelial carcinoma*	
	n	%	n	%
No	467	77%	559	92%
Yes	141	23%	49	8%

### 3.2 Level 2 – Satisfaction

246 of the 249 participants who completed the entire program responded to a satisfaction survey. The overall recommendation rate was very high: 94.3% of participants would take a course like this again, and 95.1% would recommend it to other professionals. Satisfaction with the course was rated as *Remarkable (4)* by 37.8% of participants and *Excellent (5)* by 43.1%, on a Likert scale of 1 to 5.

All participants rated the experience and format of the E-PIMUC program very positively, selecting *Agree (4)* or *Strongly Agree (5)* for all criteria, except when rating the usefulness of the mobile version. Below are the detailed survey results:

On the content and level:

• 96% considered that the course provided them with new knowledge.
• 91% agreed that the content covers the right topics.
• 93% considered that the content is up to date with current scientific evidence.
• 92% considered that the depth of knowledge in the learning pills is adequate.
• 92% thought that the duration of the learning pills is sufficient to achieve the learning objectives.
• 92% considered that the content was presented appropriately and easy to understand.

On the methodology and format:

• 88% considered that the video format supported their learning.
• 80% considered that they learn more with videos than text.
• 91% rated the methodology positively.
• 89% agreed that the bitesize content supported them to move at their own pace.
• 92% said that the overall format was beneficial for learning.

On platform usability:

• 40% felt that the mobile version helped them complete the course (37% did not use the mobile version).
• 90% felt that the course is easy to navigate.

### 3.3 Level 3 – Learning

We used a multiple-choice test to evaluate participants’ pre (baseline) and post declarative knowledge. Table 6 summarizes the mean pre and post test scores by professional profile. The average knowledge gain is statistically significant across all groups, demonstrating a substantial enhancement in participants’ understanding upon course completion.

**TABLE 6:** Participants’ declarative knowledge before and after the course, by professional profile (Level 3. Learning: declarative knowledge).

Professional profile	Knowledge score before the course (SD)	Knowledge score after the course (SD)	*p*-value
Nurse (n = 187)	4.83 (2.38)	8.93 (0.81)	*<0.001*
Physician (n = 35)	5.35 (2.50)	9.09 (0.57)	*<0.001*
Researcher (n = 8)	4.27 (2.65)	8.8 (0.77)	*<0.001*
Pharmacist (n = 19)	4.91 (2.74)	9.04 (0.65)	*<0.001*

Research participants showed the greatest mean increase in knowledge, with an average improvement of 4.53 points. The substantial improvements observed across different professional profiles showcase the value of the course and its positive impact on professional development and knowledge acquisition.

### 3.4 Level 5 – Performance

Participants were sent a self-perception questionnaire to evaluate how successfully they applied their new knowledge and skills in their professional practice, using a scale of 1 to 5. When we compared the pre and post scores, we were able to infer that participants’ confidence and knowledge of immunotherapy increased significantly. The average improvement of 1.4 points indicates a substantial enhancement in participants’ perceived abilities and understanding. This has important implications for professional practice, suggesting that the knowledge and skills participants acquired translated into increased confidence and competence when using immunotherapy in their respective roles.

Table 7 presents the mean pre and post scores for each item in the self-perception questionnaire. Higher scores indicate greater confidence and proficiency in specific practice related to immunotherapy.

**TABLE 7:** Differences in self-perceived performance before and after the course, by professional profile (Level 5. Performance).

	Mean pre (SD)	Mean post (SD)	*p*-value
Nurses: *pre = 467 answers/post = 185 answers*			
I feel confident assisting my immunotherapy patients	2.7 (1.2)	3.9 (0.9)	<0.001
I feel confident explaining immunotherapy to my patients	2.6 (1.3)	3.9 (1.0)	
I am up to date on the indications and use of immunotherapy in urothelial cancer patients	2.4 (1.2)	3.9 (0.9)	
I am familiar with the clinical guidelines on adverse effects related to immunotherapy and of its use in managing urothelial tumors	2.4 (1.2)	3.8 (1.0)	
Physicians: *pre = 85 answers/post = 35 answers*			
I feel confident assisting my immunotherapy patients	2.9 (1.2)	3.8 (1,1)	<0.001
I feel confident explaining immunotherapy to my patients	3.1 (1.2)	4.0 (0.8)	
I am up to date on the indications and use of immunotherapy in urothelial cancer patients	2.7 (1.2)	4.1 (0.8)	
I am familiar with the clinical guidelines on adverse effects related to immunotherapy and its use in managing urothelial tumors	2.7 (1.2)	3.9 (0.9)	
Researchers: *pre = 19 answers/post = 8 answers*			
I am up to date on the different preclinical models in translational research in immunotherapy	2.5 (1.4)	4.5 (0.8)	<0.003
I am familiar with the biological and molecular mechanisms underlying the action of checkpoint inhibitors/immunotherapy in urothelial cancer	2.9 (1.4)	4.5 (0.9)	
I am able to work with preclinical models for investigating new drugs and/or biomarkers related to immunotherapy in urothelial cancer	2.3 (1.2)	4.5 (0.8)	
I am up to date on the different immunotherapy drugs currently under preclinical investigation in urothelial cancer	2.3 (1.2)	4.8 (0.5)	
Pharmacists: *pre = 37 answers/post = 18 answers*			
I am aware of the toxicities associated with immunotherapy	2.9 (0.9)	3.9 (1.0)	<0.001
I am aware of the recommended treatments in case of toxicity	2.8 (1.0)	3.8 (1.0)	
I am up to date on the indications and use of immunotherapy in urothelial cancer patients	2.8 (0.9)	3.9 (1.0)	
I am familiar with the clinical guidelines on adverse effects related to immunotherapy and its use in managing urothelial tumors	2.8 (1.0)	4.0 (1.0)	

We observed statistically significant differences (*p* < 0.005) between pre and post questionnaire answers across all professional profiles. Our detailed analysis revealed the following:

Nurses: overall, nurses reported a significant improvement (*p* < 0.001) in their knowledge and confidence in assisting immunotherapy patients and explaining the treatment to them. They were also more familiar with clinical guidelines and indications for urothelial cancer treatment after the course.

Physicians: this group reported increased confidence in assisting immunotherapy patients and explaining the treatment to them. They also significantly improved (*p* < 0.001) their knowledge of indications and clinical guidelines for urothelial cancer treatment.

Researchers: these participants reported a significant improvement (*p* < 0.003) across all areas including knowledge of preclinical models, biological mechanisms, and immunotherapy drugs for urothelial cancer.

Pharmacists: this group reported a significant increase in awareness (*p* < 0.001) of toxicities associated with immunotherapy, and of the indications and guidelines for its use in urothelial cancer.

Though self-perceived performance assessments are subjective, they nonetheless provide valuable insights by exploring participants’ perspectives and beliefs about their abilities. This gives a meaningful indication of growth and confidence.

### 3.5 Benefit in the workplace

**FIGURE 2:**
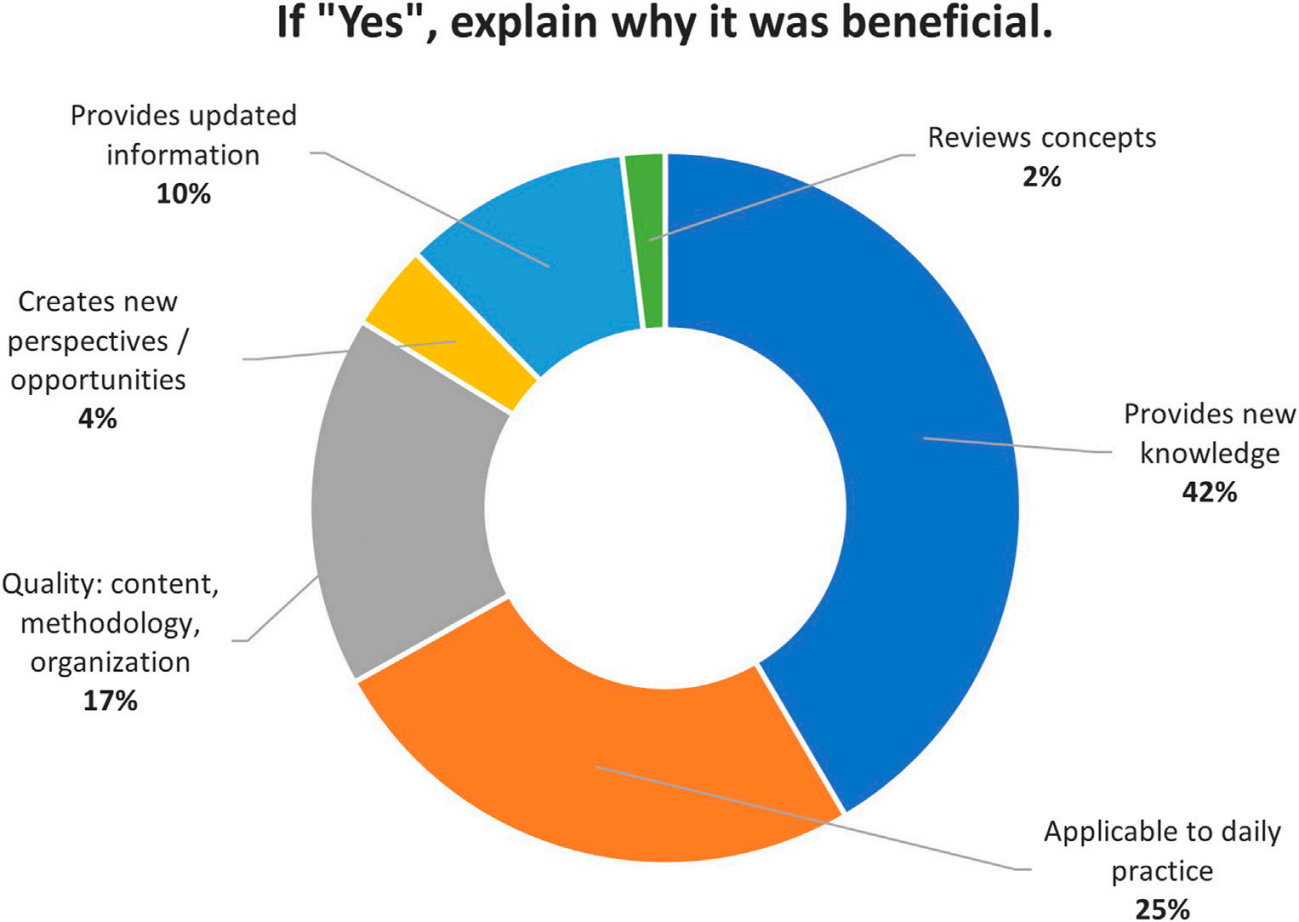
Participants’ reasons for a positive response on the perceived benefits of the course.

**FIGURE 3:**
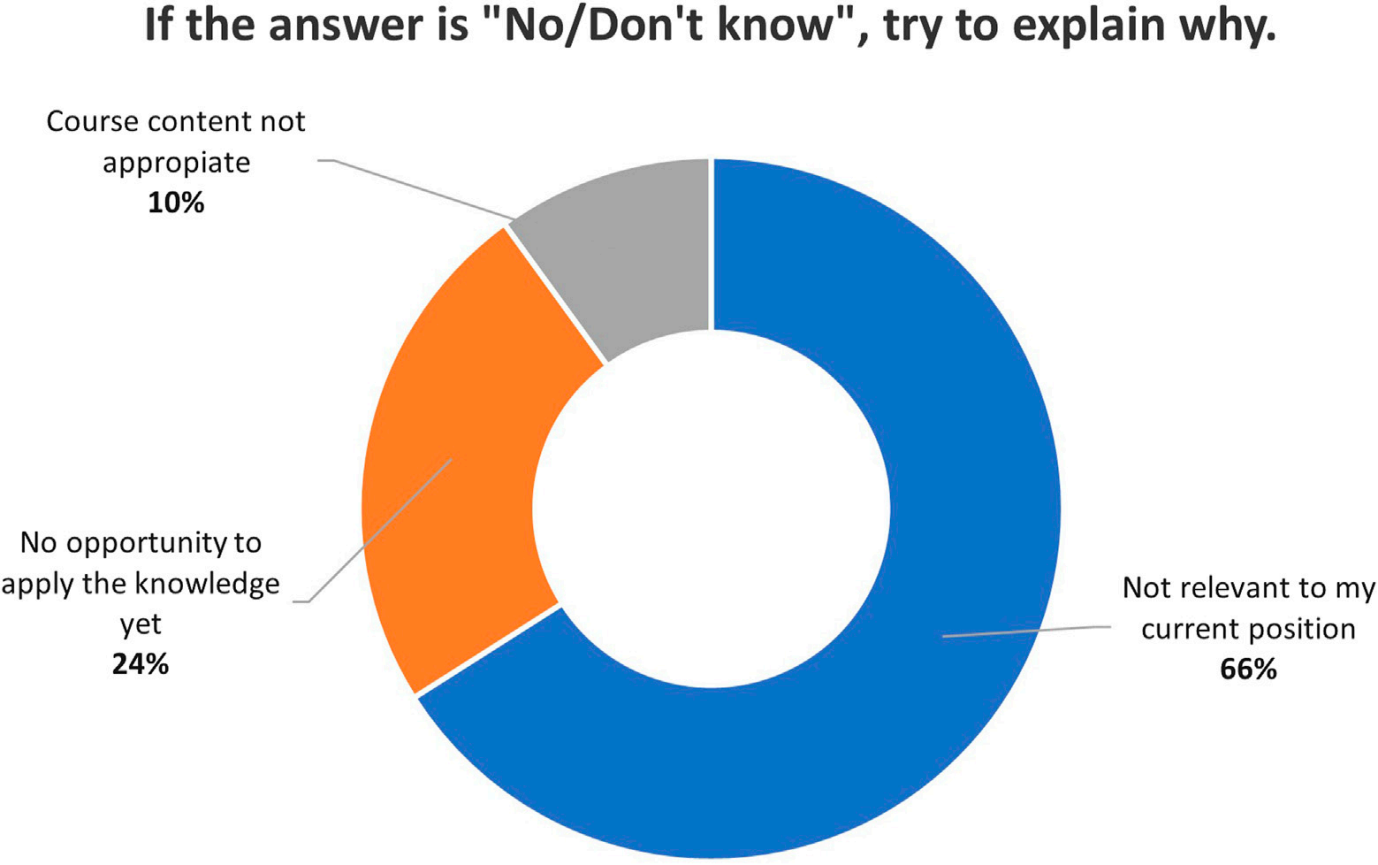
Participants’ reasons for a negative or uncertain response on the perceived benefits of the course.

Participants were overwhelmingly positive when asked about the overall benefits of the course to their work. Across all professional profiles, 85% found the course to be beneficial, only 4% responded negatively, and 10% were uncertain about the benefits. Figures 2, 3 present the reasons (when provided) for participants’ responses on the perceived benefits of the course.

## 4 Discussion

The rapid evolution and implementation of immunotherapy for urothelial cancer generated a critical need for specialized training programs for 1) healthcare professionals involved in the clinical management of patients and 2) translational researchers in this branch of oncology. Advancements such as these should be supported with targeted educational initiatives to ensure healthcare providers and researchers remain abreast of the latest developments. This, in turn, supports improved patient outcomes and advances the field as a whole.

The E-PIMUC program provided comprehensive continuing medical education and training for urothelial cancer management. It enhanced healthcare professionals’ knowledge across various dimensions of urothelial cancer treatment using a flexible and accessible microlearning format comprising learning pills, webinars, and a patient colloquium. This is similar to the format used in other studies (Bannister et al., 2020; Safavi et al., 2022). While other online educational programs for healthcare providers have explored the clinical management of immune checkpoint inhibitors in urothelial cancer (Gitzinger et al., 2021; Gitzinger et al., 2022), to the best of our knowledge the E-PIMUC program was the first to successfully employ a microlearning-based methodology.

E-PIMUC participation rates were high: over 80% of enrolled participants actively engaged with the content. This speaks to the relevance and value of the program for healthcare professionals seeking to expand their knowledge in urothelial cancer management. The diversity of participants’ professional profiles suggests that the program had broad appeal. Interestingly, E-PIMUC attracted a significant number of nursing professionals. This is promising, since nurses play a pivotal not only in administering treatments but in educating patients and managing adverse effects related to new cancer treatments, including immunotherapy.

To enable measurable knowledge acquisition and accountability for educational outcomes, microlearning initiatives should integrate evaluation at the point where participants acquire new knowledge (Bannister et al., 2020). E-PIMUC participants were assessed after each module, rather than at the end of the entire program. Pre-post testing scores show that participants substantially increased their knowledge. This aligns with other studies that reported a notable increase in clinicians’ confidence and intent to use systemic immunotherapy to treat bladder cancer; the increase was particularly significant among practitioners in academic medical centers, surging from 25% to over 70% (Gitzinger et al., 2021).

The E-PIMUC program directly enhanced participants’ understanding of urothelial cancer management and the role of immunotherapy in treating this disease. The self-perceived improvement in performance further suggests that the program had a positive impact on participants’ ability to apply their knowledge and skills in their respective professional roles. The successful integration of patient perspectives via the virtual colloquium reflects the program’s patient-centered approach–this form of inclusive dialogue between patients and healthcare professionals contributes to a holistic understanding of urothelial carcinoma management and demonstrates a commitment to addressing patients’ needs, concerns, and expectations.

The main limitations of our study include the reliance on self-reported rather than objective assessments, and the lack of long-term benefit reporting or impact assessment, with evaluations limited to relatively short-term outcomes. Potential barriers to implementing similar programmes in other settings include adaptation of content to specific situations in other countries, such as underdeveloped countries, language adaptation, and technological adaptations for online training, such as the availability of internet and computers in rural areas. Future editions of the E-PIMUC program may be launched to continue supporting healthcare professions working in immunotherapy and bladder cancer.

## 5 Conclusion

The E-PIMUC program equipped participating healthcare professionals with cutting-edge, multidisciplinary knowledge of immunotherapy in urothelial cancer treatment, including translational research and precision medicine. E-PIMUC also addressed patient management challenges, and the complementary colloquium empowered patients through education. The high participation rates, positive satisfaction scores, significant increase in knowledge as evidenced by pre-post testing, and the endorsement of regulatory bodies and professional associations, all attest to E-PIMUC’s success in delivering quality education and training in the field of urothelial cancer.

## Data Availability

The raw data supporting the conclusions of this article will be made available by the authors, without undue reservation.

## Footnotes

1 Supported by Pfizer grant IGCL 34096003.

2 Hosted on www.e-oncologia.org.

3 Entry number 09/033415-MD. https://canalsalut.gencat.cat/ca/professionals/formacio/continuada/acreditacio/index.html#googtrans (ca|es)

4
https://www.sogug.es/home

5
https://seom.org/
